# Prevalence, Risk Factors and Outcomes of Neck, Shoulders and Low-Back Pain in Secondary-School Children

**Published:** 2019-03-26

**Authors:** Houda Ben Ayed, Sourour Yaich, Maroua Trigui, Mariem Ben Hmida, Maissa Ben Jemaa, Achraf Ammar, Jihene Jedidi, Raouf Karray, Habib Feki, Yosra Mejdoub, Mondher Kassis, Jamel Damak

**Affiliations:** ^1^ Community Health and Epidemiology Department, Hedi Chaker University Hospital, University of Sfax, Tunisia; ^2^ Preventive medicine and hygiene department, Hedi Chaker University Hospital, University of Sfax, Tunisia; ^3^ High institute of sport and physical education, University of Sfax, Tunisia

**Keywords:** Adolescents, Musculoskeletal pain, Risk factors, Schools

## Abstract

**Background:** Musculoskeletal pain (MSP) is a public health problem among school-adolescents. This study aimed to identify the prevalence, risk factors and consequences of neck, shoulders and low-back pain among school-adolescents.

**Study design:** A cross-sectional study.

**Methods:** School-adolescents aged from 12 to 18 years between October 2017 and February 2018 in South of Tunisia were recruited. Eligible participants were randomly selected and were asked to respond a four-section questionnaire. Factors independently associated with MSP were determined through multivariate logistic regression analysis.

**Results:** Among 1221 enrolled subjects, shoulders, low-back and neck pain were reported in 43%, 35.8% and 32%, respectively. Multivariate analysis showed that independent risk factors of neck pain were female gender (Adjusted odds ratio AOR=1.55; *P*=0.002), using computer ≥4 hours/week (AOR=1.50; *P*=0.010), too low desk (AOR=2.30; *P*<0.001) and carrying schoolbag ≥60 minutes (AOR=1.58; *P*=0.008). Female gender (AOR=3.30; *P*<0.001), BMI ≥25 Kg/m2 (AOR=1.6; *P*=0.018), playing videogames ≥2 hours/day (AOR=2.37; *P*<0.001) and schoolbag to body weight≥10% (AOR=1.46; *P*=0.026) were independently associated with shoulders pain. For low back-pain, independent risk factors were high-school grade (AOR=2.70; *P*<0.001), playing videogames ≥2 hours/day (AOR=1.83; *P*<0.001), watching TV≥12 hours/week (AOR=1.5; *P*=0.016), too low seat backrest (AOR=1.4; *P*=0.005) and too far seat-to-black (board) distance (AOR=1.5; *P*=0.041). Schooladolescents consumed drugs for MSP in 19.5%, had sleep disturbance in 34% and aggressive behaviors in 22.8%.

**Conclusions:** The prevalence of MSP was substantially high among school-adolescents and their associated risk factors included sociodemographic factors, leisure activities and classroom furniture. An ergonomic specific and behavior-based school program is urgently needed.

## Introduction

Musculoskeletal pain (MSP) is a public health problem in both genders and in all age groups all around the world ^1,2^. It is considered as a frequent and costly occupational health problem in both developed and developing countries ^3^. MSP may cause considerable personal discomfort due to pain, disability, impaired quality of life and time lost from work in adulthood. According to the WHO Global Burden of Disease study, neck pain and other musculoskeletal diseases were ranked at 4^th^ and 10^th^ respectively for 15–19-year-old, among all health conditions for years lived with disability, which is higher than well-known adolescent public health problems such as asthma, alcohol use, drug use, and road injury ^4^. Recent evidence has shown that MSP is very common among school-adolescents, with a reported incidence of neck, shoulder and back pain ranging from 7% to 74% ^5–8^. The differential diagnosis of MSP in adolescents-age group is broad and the most common etiologies are classically related to musculoskeletal overuse or trauma ^7^. The development of MSP in adolescents is of concern since it increases the risk of developing chronic MSP in adulthood ^9^. The recurring and increasing rates of MSP generates significant costs for governments, making the knowledge of its potential risk factors and implementation of effective new preventive reforms urgently needed. Studies on low back pain have been widely undertaken, notably in developed countries ^10–12^. However, few researches have evaluated simultaneously neck, shoulders and low back pain in school-aged adolescents ^13^. From this perspective, we aimed, through this study, to primarily identify the prevalence of neck, shoulders and low-back pain occurrence among school-adolescents, secondly to investigate their potential risk factors and finally to assess their consequences on physical and social life.

## Methods

### Study design and settings

We conducted a questionnaire base cross-sectional study among school-adolescents of secondary school grade in the governorate of Sfax, from October, 15^th^, 2017 to 15^th^, February, 2018. Sfax governorate is situated in Southern Tunisia and accounted for 8.7% of the total Tunisian population ^14^. It is a coastal region, with almost 73605 school-adolescents, and attracts students from the neighboring rural regions. In Tunisia, secondary school grade is divided into two periods: first 3 years of basic education including adolescents aged from 12 to 14 years and 4 years of high school hosting children between 15 and 18 years. High school includes common core (4^th^ and 5^th^ years) and specialized studies (6^th^ and 7^th^ years). By the end of the 7^th^ year, school-adolescents are asked to pass a national graduating exam, after which they would be able to attend college for higher education.

### Sampling procedures

Sample size calculation was carried out assuming an overall prevalence of back pain of 30% based on data from previous studies ^15^. The minimum sample size needed was 888 subjects, using a precision of 3%. Seven strata of included adolescents were selected proportionally to each level of study. A stratified three-step cluster sampling procedure was used to obtain a representative sample of school-adolescents in the study area. In the ﬁrst stage, three educational urban districts and three educational rural districts were chosen (as strata), and then in the second stage, a total of 6 schools were selected randomly from eligible major public secondary schools of these districts. Subsequently, within each eligible school, we selected randomly 2 classes per grade (from 1^st^ to 7^th^ year). On average, there were 20 students per class. Finally, the study participants were recruited exhaustively from the eligible classes. Finally, a sample of 1221 school-adolescents who were in grade one through 7 of secondary school completed the questionnaire and were recruited for the study. Permission to approach schools in the study area was obtained from the Regional Directory of Education, the Tunisian Ministry of Education and the school authorities involved.

### Inclusion criteria

The study was carried out during weekdays and regular school hours during the study period. All students who were in the grades identified were eligible to participate in the study. Adolescents who were unable to stand on the scale unaided or used medical devices, such as plaster casts or prostheses, were excluded from the study. Those who were unable to communicate or unable to respond to the questionnaire due to medical and cognitive conditions were also excluded from participation. For the purposes of this study, we did not include patients reporting pain associated with trauma or congenital and systemic diseases.

### Data collection

Eligible participants were asked to respond a four-section questionnaire distributed at the beginning of the course, based on their own perceptions. It included demographic and socioeconomic data, behavioral factors, school-related variables and musculoskeletal complaints. The questionnaire was reviewed, checked and approved by experts, and revised after a pilot study. The investigators were trained based on a standardized protocol of data collection procedures previously established to minimize possible variability intra and inter evaluator.

Demographic and socioeconomic data included age, gender, school grade, residency, family income and parents’ educational level. Behavioral factors dealt with time spent using electronic devices and playing sport. Further investigation explored the use of school-related items, which were selected based on a literature review and prior knowledge of potential factors that may inﬂuence musculoskeletal pain in school-adolescents, including self-perceived classroom furniture/layout design, homework, supplementary tutoring and school bag carriage variables. The fourth part of the questionnaire described the musculoskeletal pain (MSP) defined as the presence of neck, shoulders or low back pain during the last 3 months. This section included standardized questions about MSP as follows: “Have you, at any time during the past month, had trouble (such as ache, pain, discomfort or numbness) in the following areas of your body?” 1) Neck, 2) shoulders, and 3) low-back area (response alternatives: yes/no). The location of these anatomic areas was demonstrated using a pre-shaded manikin picture showing the pain location and the modified standardized Nordic Musculoskeletal Questionnaire (NMQ)^16^. Those adolescents who reported pain in any of these areas were asked to quantify its severity using the Visual Analogue Scale, graded from 0 (no pain) to 10 (worst pain). Respondents were asked to indicate the effects of these complaints on medical care, their physical performance and on their social life.

### Anthropometric measurements

The study was carried out on an unannounced day for adolescents so that they could not alter their schoolbag weight. Height, body weight and schoolbag weight of school-adolescents were measured by trained personnel according to the World Health Organization (WHO) guidelines ^17^. Height was measured in centimeters to the nearest 0.5 centimeter (cm) using a measuring scale equipped with a sliding head component marked on a vertical wall. Body weight was determined in Kilograms (Kg) using a calibrated digital balanced to the closest 0.1 kg. Prior to use, all scales were calibrated with a standard weight. Body mass index (BMI) was calculated using the formula weight (kg)/height (m^2^). Subsequently, school-bag to body weight ratio was calculated and represented as percentage.

### Statistical Analysis

Statistical analysis was performed using SPSS.24. The results of quantitative variables were presented as mean ± standard deviation (SD) or median and interquartile range (IQR). Qualitative variables were presented as percentages. Student T test was used to compare two means and Chi-square test was performed for categorical variables in independent samples. Then, All variables signiﬁcant at p<0.05 in the logistic univariate analysis were entered into three separate multivariate models using a logistic binary regression (backward stepwise) [adjusted Odds Ratio (AOR); CI_95%_ (Confidence Interval _95%_), p] in order to determine the independent factors associated with a neck, shoulders and back pain, after adjusting on the confounding variables. *P* values lower than 0.05 were considered statistically significant.

## Results

### School-adolescents characteristics

The questionnaire was completed by all 1221 school-adolescents. There were 492 boys (40.3%). The sex ratio (male/female) was 0.67. The mean age of participants was 15.6 ± 2 years. Of all adolescents, 633 cases (51.8%) were aged 16 years and above. According to secondary school grade, basic education accounted for 45.7% of cases. Recruited adolescents came from a rural area in 801 cases (65.6%). The adolescents’ family had mostly a middle income (909 cases; 74.4%). The most common education level of parents was high-school (525 cases; 43%) for fathers and illiteracy/primary school for mothers (529 cases; 43.3%). The mean body weight of school-adolescents was 59 ±14.4 Kg and the mean height was 166 ±9.67 cm. There were 232 adolescents with a BMI ≥25 Kg/m^2^ (19%). The mean schoolbag weight for all of the adolescents was 3 ±1.3 kg. The mean school bag weight as percentage of body weight was 5.4 ± 2.8%. The most popular method of carrying a schoolbag was on both shoulders (57.2%).

### Neck, shoulders and low-back complaints

Overall, 525 school-adolescents reported shoulder pain (43%), while back and neck pain were reported in 437 cases (35.8%) and 391 cases (32%), respectively. Of all participants, 194 cases (15.9%) had complaints in two different sites and 83 cases (6.8%) had complaints in three sites simultaneously. The mean value of the pain severity in the neck, shoulder and low- back areas was 4.8 ± 2, 4.3 ± 2.4 and 4.6 ± 2.3 respectively.

### Basic demographics, individual and leisure activities risk factors

Results of logistic univariate analysis showed that girls experienced neck (OR=1,7; p<0.001) and shoulders pain (OR=2.7; p<0.001) more than boys. Age ≥16 years was significantly associated with neck (OR=1.5; p=0.001) and low back pain (OR=1.7; p<0.001). Moreover, the proportion of adolescents with BMI≥25 Kg/m^2^ was significantly higher among neck (OR=1.6; p=0.015), shoulders (OR=1.65; p=0.004) and low back pain adolescents (OR=1.7; p=0.003). High school grade including common core and specialized studies were respectively more susceptible to neck (OR=1.38; p=0.024 and OR=1.8; p<0.001) and low back pain (OR=1.8; p<0.001 and OR=2.2; p<0.001). Adolescents living in urban areas had significantly more low back pain complaints than rural areas (OR=1.3; p=0.026).

School-adolescents living with low or middle-income families were more likely to suffer from shoulders (OR=1.6; p=0.046) and low back pain (OR=1.67; p=0.025).

Furthermore, leisure activities such as using computer for more than 4 hours was significantly associated with neck (OR=1.54; p=0.003) and low back pain (OR=1.56; p=0.002). Adolescents playing videogames two hours and above per day had significantly more shoulders (OR=1.6; p=0.001) and low back complaints (OR=1.61; p=0.001), while watching TV for 12 hours or more per week was significantly associated with low-back pain (OR=1.5; p=0.01).

On the other hand, playing sport for one to three hours per week reduced the risk of neck (OR=0.71; *P*=0.036), shoulders (OR=0.52; *P*<0.001) and low-back pain occurrence (OR=0.71; *P*=0.046) in school-adolescents (Table 1). The same findings were applied to higher education level of the father (high school or college degree), with respective OR of 0.7 (*P*=0.017), 0.72 (*P*=0.038) and 0.72 (*P*=0.040) for neck, shoulders and low back pain, respectively. As for mother education level, high school level was significantly associated with lower risk of shoulders pain (OR=0.72; *P*=0.016).

**Table 1:** Neck, shoulders and low-back complaints according to demographic and individual factors, socioeconomic level and behavioral factors: results of univariate logistic regression analysis

** Variables **	** Total (n=1221)**	** Neck pain (n=391; 32%)**			** Shoulders pain (n=525; 43%)**			**Back pain (n=437; 35.8%) **		
	**n (%)**	**n (%) **	**OR (95% CI)**	**P value **	**n (%) **	**OR (95% CI)**	**P value**	** n (%)**	**OR (95% CI)**	** P value**
Gender Male	492 (40.3)	124 (25.2)	1.00		142 (28.9)	1.00		164 (33.3)	1.00
Female	729 (59.7)	267 (36.6)	1.70 (1.30, 2.20)	0.001	383 (52.5)	2.70 (2.10, 3.48)	0.001	273 (37.4)	1.17 (0.90, 1.52)	0.140
Age groups (yr) 12-16	588 (48.2)	160 (27.2)	1.00		249 (42.3)	1.00		174 (29.6)	1.00
>16	633 (51.8)	231 (36.5)	1.50 (1.20, 1.96)	0.001	276 (43.6)	1.03 (0.83, 1.30)	0.600	263 (41.5)	1.70 (1.30, 2.10)	0.001
BMI (kg/m^2^) <18	271 (22.1)	72 (26.6)	1.00	0.048	112 (41.3)	1.00	0.001	93 (34.3)	1.00	0.001
18-25	701 (57.2)	236 (32.9)	1.30 (0.97, 1.80)	0.072	296 (41.2)	0.94 (0.70, 1.20)	0.660	244 (34.0)	0.90 (0.70, 1.20)	0.560
>25	229 (20.7)	83 (35.8)	1.60 (1.10, 2.30)	0.015	117 (50.4)	1.65 (1.16, 2.30)	0.004	100 (43.1)	1.70 (1.10, 2.40)	0.003
Secondary school grade Basic education (first 3 yr)	558 (45.7)	150 (26.9)	1.00	0.001	231 (41.4)	1.00	0.380	152 (27.2)	1.00	0.001
High school Common core (4-5th years)	392 (32.1)	132 (33.7)	1.38 (1.10, 1.80)	0.024	168 (42.9)	1.06 (0.80, 1.30)	0.650	161 (41.1)	1.80 (1.40, 2.40)	0.001
Specialized terminal studies (6-7th yr)	271 (22.2)	109 (40.2)	1.80 (1.34, 2.48)	0.001	126 (46.5)	1.23 (0.90, 1.60)	0.160	124 (45.8)	2.20 (1.66, 3.00)	0.001
Residency Rural	801 (65.6)	257 (32.1)	1.00		344 (42.9)	1.00		269 (33.6)	1.00
Urban	420 (34.4)	134 (31.9)	0.99 (0.77, 1.20)	0.940	181 (43.1)	1.01 (0.80, 1.20)	0.960	168 (40.0)	1.30 (1.10, 1.60)	0.026
Socioeconomic level Family financial situation ($)
High (>1000)	112 (9.2)	38 (33.9)	1.00	0.690	39 (34.8)	1.00	0.120	28 (25.0)	1.00	0.012
Middle (200-1000)	909 (74.4)	285 (31.4)	1.03 (0.6, 1.63)	0.990	393 (43.2)	1.60 (1.10, 2.60)	0.046	325 (35.8)	2.17 (1.30, 3.60)	0.003
Low (<200)	200 (16.4)	68 (34.0)	0.89 (0.6, 1.34)	0.580	93 (46.5)	1.40 (0.90, 2.10)	0.090	84 (42.0)	1.67 (1.10, 2.61)	0.025
Father education level Illiterate/primary school	419 (34.3)	151 (36.0)	1.00	0.059	203 (48.4)	1.00	0.021	167 (39.9)	1.00	0.080
High school	525 (43.0)	151 (28.8)	0.7 (0.54, 0.94)	0.017	210 (40.0)	0.70 (0.54, 0.91)	0.009	180 (34.3)	0.78 (0.60, 1.00)	0.070
College degree	277 (22.7)	89 (32.1)	0.84 (0.6, 1.15)	0.290	112 (40.4)	0.72 (0.52, 0.83)	0.038	90 (32.5)	0.72 (0.52, 0.90)	0.040
Mother education level Illiterate/primary school	529 (43.3)	168 (31.8)	1.00	0.940	249 (47.1)	1.00	0.040	180 (34.0)	1.00	0.300
High school	435 (35.6)	142 (32.6)	1.00 (0.79, 1.36)	0.770	171 (39.3)	0.72 (0.52, 0.94)	0.016	155 (35.6)	1.1 (0.82, 1.40)	0.600
College degree	257 (21.0)	81 (31.5)	0.98 (0.71, 1.36)	0.940	105 (40.9)	0.77 (0.57, 1.10)	0.100	102 (39.7)	1.27 (0.94, 1.73)	0.100
Hours per week playing sport <1	237 (19.4)	88 (37.1)	1.00	0.100	119 (50.2)	1.00	0.001	99 (41.8)	1.00	0.100
1-3	630 (51.6)	187 (29.7)	0.71 (0.52, 0.92)	0.036	284 (45.1)	0.81 (0.60, 1.10)	0.170	218 (34.6)	0.73 (0.54, 1.10)	0.045
>3	354 (29.0)	116 (32.8)	0.82 (0.58, 1.16)	0.270	122 (34.5)	0.52 (0.37, 0.73)	0.001	120 (33.9)	0.71 (0.51, 1.10)	0.046
Hours per week using a computer <1	613 (50.2)	186 (30.3)	1.00	0.001	271 (44.2)	1.00	0.690	201 (32.8)	1.00	0.007
1-4	305 (25.0)	83 (27.2)	0.85 (0.63, 1.16)	0.320	127 (41.6)	0.90 (0.68, 1.18)	0.460	105 (34.4)	1.10 (0.80, 1.43)	0.620
>4	303 (24.8)	122 (40.3)	1.54 (1.16, 2.06)	0.003	127 (41.9)	0.91 (0.68, 1.20)	0.510	131 (43.2)	1.56 (1.17, 2.10)	0.002
Hours per week watching TV <3	552 (45.2)	182 (33.0)	1.00	0.068	238 (43.1)	1.00	0.780	186 (33.7)	1.00	0.029
3-12	441 (36.1)	125 (28.3)	0.80 (0.61, 1.01)	0.110	185 (42.0)	0.95 (0.74, 1.20)	0.710	152 (34.5)	1.03 (0.79, 1.34)	0.790
>12	228 (18.7)	84 (36.8)	1.18 (0.85, 1.60)	0.300	102 (44.7)	1.10 (0.78, 1.40)	0.670	99 (43.4)	1.50 (1.10, 2.07)	0.010
Hours per day playing videogames <1	649 (52.2)	212 (32.7)	1.00	0.490	266 (41.0)	1.00	0.001	212 (32.7)	1.00	0.002
1-2	249 (20.4)	72 (28.9)	0.80 (0.60, 1.15)	0.280	89 (35.7)	0.80 (0.52, 1.10)	0.150	83 (33.3)	1.03 (0.75, 1.40)	0.840
>2	323 (26.5)	107 (33.1)	0.83 (0.76, 1.30)	0.880	170 (52.6)	1.60 (1.20, 2.10)	0.001	142 (44.0)	1.61 (1.20, 2.10)	0.001

N: Number; OR: Odds Ratio; 95% CI: 95% confidence interval; yr: Years; BMI: Body Mass Index

### School-related risk factors

Classroom furniture and layout design associated with both neck and low back pain included too low seat (OR=1.63; *P*<0.001 and OR=1.5; *P*=0.001, respectively), too low seat backrest (OR=1.62; *P*<0.001 and OR=1.6; *P*<0.001, respectively) as well as too narrow seat (OR=1.4; *P*=0.034 and OR=1.6; *P*=0.004, respectively). A too low desk was significantly more frequent in school-adolescents reporting neck pain (OR=2.4; *P*<0.001), while a too near seat-to (black) board distance was statistically more common among shoulders pain adolescents (OR=1.3; *P*=0.04) (Table 2). Too much homework increased significantly the risk of neck pain (OR=1.4; *P*=0.021), while supplementary tutoring increased the risk of back pain complaints (OR=1.3; *P*=0.037).

**Table 2:** School-related factors and self-reported presence of neck, shoulder and back pain: results of univariate logistic regression analysis

**Variables **	**Total n=1221 (%)**	** Neck pain (n=391; 32%) **			**Shoulders pain (n=525; 43%)**			**Back pain (n=437; 35.8%)**		
** Classroom furniture/layout design **		**n (%)**	**OR (95% CI)**	**P value**	**n (%) **	**OR (95% CI)**	**P value**	** n (%) **	**OR (95% CI)**	** P value **
Just right	852 (69.8)	250 (29.3)	1.00	0.001	375 (44.0)	1.00	0.210	282 (33.1)	1.00	0.004
Too high	55 (4.5)	14 (25.5)	0.82 (0.40, 1.50)	0.539	27 (49.1)	1.20 (0.70, 2.10)	0.460	18 (32.7)	0.98 (0.55, 1.70)	0.950
Too low	314 (25.7)	127 (40.4)	1.63 (1.20, 2.00)	0.001	123 (39.2)	0.80 (0.62, 1.10)	0.140	137 (43.6)	1.50 (1.20, 2.00)	0.001
Seat backrest height Just right	643 (52.7)	177 (27.5)	1.00	0.001	277 (43.1)	1.00	0.640	201 (31.3)	1.00	0.001
Too high	69 (5.7)	20 (29.0)	1.06 (0.60, 1.80)	0.790	26 (37.7)	0.80 (0.50, 1.30)	0.390	21 (30.4)	0.96 (0.56, 1.65)	0.880
Too low	509 (41.7)	194 (38.1)	1.62 (1.20, 2.00)	0.001	222 (43.6)	1.10 (0.80, 1.30)	0.850	215 (42.2)	1.60 (1.26, 2.00)	0.001
Seat width Just right	903 (74.0)	278 (30.8)	1.00	0.098	386 (42.7)	1.00	0.510	301 (33.1)	1.00	0.008
Too wide	124 (10.2)	38 (30.6)	0.99 (0.66, 1.50)	0.970	59 (47.6)	1.20 (0.80, 1.70)	0.300	50 (40.3)	1.35 (0.92, 1.98)	0.120
Too narrow	194 (15.9)	75 (38.7)	1.40 (1.10, 1.90)	0.034	80 (41.2)	0.94 (0.68, 1.30)	0.700	86 (44.3)	1.60 (1.10, 2.10)	0.004
Desk height Just right	911 (74.6)	258 (28.3)	1.00	0.001	385 (42.3)	1.00	0.270	312 (34.2)	1.00	0.150
Too high	73 (6.0)	16 (21.9)	0.71 (0.40, 1.26)	0.240	28 (38.4)	0.85 (0.52, 1.40)	0.510	29 (39.7)	1.26 (0.77, 2.00)	0.340
Too low	237 (19.4)	117 (49.4)	2.40 (1.80, 3.30)	0.001	112 (47.3)	1.20 (0.90, 1.60)	0.160	96 (40.5)	1.30 (0.97, 1.75)	0.073
Seat, to, (black) board distance Just right	873 (71.5)	292 (33.4)	1.00	0.220	373 (42.7)	1.00	0.034	310 (35.5)	1.00	0.100
Too near	209 (17.1)	58 (27.8)	0.76 (0.50, 1.00)	0.110	103 (49.3)	1.30 (1.10, 1.70)	0.040	67 (32.1)	0.85 (0.62, 1.18)	0.340
Too far	139 (11.4)	41 (29.5)	0.83 (0.56, 1.20)	0.350	49 (35.3)	0.73 (|0.50, 1.10)	0.098	60 (43.2)	1.37 (0.95, 1.98)	0.083
Homework Just right/not enough	960 (78.6)	292 (30.4)	1.00		400 (41.7)	1.00		339 (35.3)	1.00
Too much	261 (21.4)	99 (37.9)	1.40 (1.10, 1.80)	0.021	125 (47.9)	1.20 (0.97, 1.60)	0.072	98 (37.5)	1.10 (0.80, 1.40)	0.500
Supplementary tutoring No	240 (19.7)	78 (32.5)	1.00		96 (40.0)	1.00		72 (30.0)	1.00
Yes	981 (80.3)	313 (31.9)	0.90 (0.70, 1.10)	0.860	429 (43.7)	1.10 (0.80, 1.50)	0.290	365 (37.2)	1.30 (1.10, 1.80)	0.037
School bag weight as % body weight 10%	1019 (83.5)	338 (32.1)	1.00		438 (41.6)	1.00		382 (36.2)	1.00
>10%	202 (16.5)	53 (31.7)	0.90 (0.70, 1.40)	0.930	87 (52.1)	1.50 (1.16, 2.14)	0.003	55 (32.9)	1.44 (1.10, 2.00)	0.019
Time spent carrying school bag (min/day) 30	277 (22.7)	69 (24.9)	1.00	0.013	96 (34.7)	1.00	0.001	82 (29.2)	1.00	0.030
30-60	357 (29.2)	117 (32.8)	1.40 (1.10, 2.00)	0.031	146 (40.9)	1.30 (0.94, 1.80)	0.100	139 (38.9)	1.50 (1.10, 2.10)	0.011
>60	587 (48.1)	205 (34.9)	1.60 (1.17, 2.20)	0.003	283 (48.2)	1.75 (1.30, 2.00)	0.001	217 (37.0)	1.40 (1.10, 1.90)	0.026
Time spent from home to school (min) 15	624 (51.1)	195 (31.3)	1.00	0.450	244 (39.1)	1.00	0.003	215 (34.5)	1.00	0.550
15-30	399 (32.7)	125 (31.3)	1.00 (0.70, 1.30)	0.970	177 (44.4)	1.24 (0.96, 1.60)	0.960	146 (36.6)	1.10 (0.84, 1.42)	0.480
>30	198 (16.2)	71 (35.9)	1.20 (0.80, 1.70)	0.220	104 (52.5)	1.72 (1.20, 2.40)	0.001	76 (38.4)	1.18 (0.85, 1.64)	0.310
Method of school bag carriage Both shoulders	699 (57.2)	193 (27.6)	1.00	0.001	274 (39.2)	1.00	0.001	244 (34.9)	1.00	0.034
One shoulder	497 (40.7)	192 (38.6)	1.60 (1.29, 2.00)	0.001	246 (49.5)	1.50 (1.20, 1.90)	0.001	190 (38.2)	1.15 (0.90, 1.40)	0.240
By hand	25 (2.0)	6 (24.0)	0.82 (0.32, 2.00)	0.690	5 (20)	0.38 (0.14, 1.00)	0.060	3 (12.0)	0.25 (0.07, 0.80)	0.027
Method of travel to/from school Bus/Car/Bike	566 (46.4)	157 (27.7)	1.00		253 (44.7)	1.00		201 (35.5)	1.00
Walk	655 (53.6)	234 (35.7)	0.69 (0.50, 0.80)	0.003	272 (41.5)	1.10 (0.90, 1.40)	0.260	236 (36.0)	0.97 (0.70, 1.20)	0.850

N: Number; OR: Odds Ratio; 95% CI: 95% confidence interval; min: minutes

As for schoolbag carriage variables, time spent carrying schoolbag for more than 60 min/day was associated with neck (OR=1.6; *P*=0.003), shoulders (OR=1.75; *P*<0.001) and low back pain (OR=1.4; *P*=0.026). Adolescents carrying schoolbag on one shoulder reported higher complaints of both neck (OR=1.6; *P*<0.001) and shoulders (OR=1.5; *P*<0.001) pain than on both shoulders, while adolescents carrying it by hand had significantly less low back complaints (OR=0.25; *P*=0.027). Schoolbag weight as percentage of body weight ≥10% increased the likelihood of reporting shoulders (OR=1.5; *P*=0.003) and low back pain (OR=1.44; *P=*0.019). Besides, time spent from home to school more than 30 minutes was statistically associated with higher proportion of shoulders pain (OR=1.72; *P*=0.003). Walking from/to school decreased significantly the risk of neck pain (OR=0.69; *P*=0.003).

### Results of multivariate analysis

Multivariate analysis showed that factors independently associated with neck pain were female gender (AOR=1.55; *P*=0.002), high school education level of the father (AOR=0.73; *P*=0.033), using computer ≥4 hours/week (AOR=1.5; *P*=0.010), too low desk (AOR=2.3; *P*<0.001), carrying schoolbag for 60 minutes or more (AOR=1.58; *P*=0.008) and on one shoulder (AOR=1.46; *P*=0.005) as well as walking from/to school (AOR=0.68; *P*=0.003).

Female gender (AOR=3.3; *P*<0.001), BMI ≥25 Kg/m^2^ (AOR=1.6; *P*=0.018), playing videogames more than 2 hours per day (AOR=2.37; *P*<0.001), too much homework (AOR=1.38; *P*=0.030), schoolbag weight as ercentage of body weight ≥10% (AOR=1.46; *P*=0.026) and carrying schoolbag ≥60 minutes/day (AOR=1.7; *P*<0.001) were independently associated with shoulders pain.

As for low back pain, independent risk factors were high school grade, notably common core (AOR=2.1; *P*<0.001) and specialized terminal studies (AOR=2.7; *P*<0.001), playing videogames for more than 2 hours per day (AOR=1.83; *P*<0.001) and watching TV for more than 12 hours per week (AOR=1.5; p=0.016). Independent school related risk factors included too low seat backrest (AOR=1.4; *P*=0.005), too far seat to black (board) distance (AOR=1.5; *P*=0.041), schoolbag weight as percentage of body weight ≥10% (AOR=1.7; *P*=0.002) and carrying schoolbag for 30 to 60 minutes (AOR=1.48; *P*=0.029). High education level of the father (college degree) (AOR=0.7; *P*=0.046) and carrying school bag by hand (AOR=0.25; *P*=0.033) were independently associated with lower risk of low back pain (Table 3).

**Table 3:** Risk factors of neck, shoulder and back pain: results of multivariate analysis

**Variables**	**AOR (95% CI)**	***P*** ** value**
**Neck pain**
Gender		
Male	1.00	
Female	1.55 (1.18, 2.00)	0.002
Father education level		
Illiterate/primary school	1.00	
High school	0.73 (0.54, 0.9)	0.033
College degree	0.78 (0.55, 1.1)	0.160
1	1.00	
1-4	0.95 (0.69, 1.30)	0.780
>4	1.50 (1.10, 2.00)	0.010
Desk height		
Just right	1.00	
Too high	0.80 (0.40, 1.40)	0.500
Too low	2.30 (1.70, 3.10)	0.001
Time spent carrying school bag (min/day)		
30	1.00	0.029
30-60	1.37 (0.94, 1.97)	0.090
>60	1.58 (1.12, 2.20)	0.008
Method of school bag carriage		
Both shoulders	1.00	0.016
One shoulder	1.46 (1.12, 1.90)	0.005
By hand	0.96 (0.36, 2.57)	0.940
Method of travel to/from school		
Bus/Car/Bike	1.00	
Walk	0.68 (0.52, 0.90)	0.003
**Shoulders pain**
Gender		
Male	1.00	
Female	3.30 (2.50, 4.40)	0.001
BMI (Kg/m^2^)		
18	1.00	0.001
18-25	0.83 (0.26, 1.14)	0.260
>25	1.60 (1.10, 2.40)	0.018
Hours per week playing games		
1	1.00	0.001
1-2	1.10 (0.78, 1.50)	0.630
>2	2.37 (1.75, 3.22)	0.001
Just right/not enough	1.00	
Too much	1.38 (1.1, 1.86)	0.030
School bag weight as % body weight		
10%	1.00	
>10%	1.46 (1.10, 2.00)	0.026
Time spent carrying school bag (min/day)		
30	1.00	0.002
30-60	1.34 (0.95, 1.90)	0.091
>60	1.70 (1.30, 2.40)	0.001
**Back pain**
High school		
Common core (4th and 5th years)	2.10 (1.60, 2.90)	0.001
Specialized terminal studies (6th and 7th)	2.70 (1.90, 3.70)	0.001
Father education level		
Illiterate/primary school	1.00	
High school	0.76 (0.57, 1.00)	0.058
College degree	0.70 (0.50, 0.90)	0.046
Hours per week playing games		
1	1.00	0.001
1-2	1.16 (0.83, 1.60)	0.370
>2	1.83 (1.34, 2.50)	0.001
Hours per week watching TV		
3	1.00	0.048
3-12	1.00 (0.80, 1.40)	0.700
>12	1.50 (1.10, 2.10)	0.016
Seat backrest height		
Just right	1.00	0.012
Too high	0.92 (0.52, 1.60)	0.770
Too low	1.40 (1.12, 1.87)	0.005
Seat, to, (black) board distance		
ust right	1.00	0.090
Too near	0.92 (0.65, 1.29)	0.640
Too far	1.50 (1.10, 2.20)	0.041
School bag weight as % body weight		
10%	1.00	
>10%	1.70 (1.20, 2.40)	0.002
Time spent carrying school bag (min/day)		
30	1.00	0.089
30-60	1.48 (1.10, 2.10)	0.029
>60	1.22 (0.88, 1.70)	0.210
Method of school bag carriage		
Both shoulders	1.00	0.090
One shoulder	1.00 (0.79, 1.33)	0.800
By hand	0.25 (0.07, 0.89)	0.033

AOR: Adjusted Odds Ratio; 95% CI: 95% confidence interval; BMI: Body Mass Index; min: minutes; For variables with 3 or more categories, the first p-value is the global p-value of the test. “-”: No statistically significant association between this variable and the dependent variable.

### Medical and social effects of musculoskeletal pain in school adolescents

School-adolescents consulted a doctor for a low back pain in 107 cases (24.5%), for a neck pain in 74 cases 18.9% and for shoulders pain in 85 cases (16.2%). Prescription of pharmacological treatment, X ray exploration, need for physical therapy and walking difficulties were mostly noted for low back pain (24%, 11.9%, 9.6% and 19% respectively). Sport practice difficulties were commonly noted in shoulders pain, while sleep disturbance was more frequently noted in neck pain (42.7%). Of all school-adolescents, 6.9% were exempted from physical education for a low back pain, 28.9% reported a decrease in school marks for a neck pain, 24.5% had peer problems for a low back pain and 8.8% reported poor communication for shoulders pain. Besides, 22.2% of patients reporting low back complaints had a need for assistance for personal daily activities. Aggressive behaviors were reported by adolescents with neck pain in 31.2%, shoulders pain in 25.7% and low back pain in 26.1% (Table 4).

**Table 4:** Effects of neck, shoulders and low-back pain on medical care, physical performance and social life in school adolescents

**Variables**	**Total** **n=978**		**Neck pain** **n=391**		**Shoulders pain** **n=525**		**Low back pain** **n=437**	
	**n**	**%**	**n**	**%**	**n**	**%**	**n**	**%**
Medical care								
Consulting a doctor	177	18.1	74	18.9	85	16.2	107	24.5
Prescription of pharmacological treatment	191	19.5	82	21.0	98	18.7	105	24.0
X ray exploration	85	8.7	35	9.0	43	8.2	52	11.9
Need for physical therapy sessions	80	8.2	32	8.2	39	7.4	42	9.6
Physical performance								
Walking difficulties	144	14.7	72	18.4	93	17.7	83	19.0
Sport practice difficulties	209	21.4	97	24.8	137	26.1	103	23.6
Sleep disturbance	333	34.0	167	42.7	181	34.5	164	37.5
Physical education exemption (sports absenteeism)	50	5.1	15	3.8	26	5.0	30	6.9
Social effects								
Decreased school marks	257	26.3	113	28.9	148	28.2	118	27.0
Peer problems	192	19.6	90	23.0	113	21.5	107	24.5
Poor communication	70	7.2	36	9.2	46	8.8	35	8.0
Need for assistance for personal daily activities	178	18.2	86	22.0	111	21.1	97	22.2
Aggressive behaviors	223	22.8	122	31.2	135	25.7	114	26.1

## Discussion

The purpose of this study was to synthesize the evidence for potential adolescent musculoskeletal pain prevalence, risk factors and main outcomes in school-adolescents. This is one of the largest studies of its kind to evaluate the magnitude of neck, shoulders or back pain among school adolescents in Southern Tunisia. Although researchers have explored numerous features of musculoskeletal, simultaneous multi-site pain assessment in adolescents was poorly addressed in the literature; this study added more evidence to the MSP debate and extended the body of knowledge through developing and low-income countries.

The occurrence of low back, neck and shoulders complaints was relatively high in our study population, ranging from 32 to 43%, which was in line with previous reports from developed countries ^18^. In a Danish study investigating the frequency of low back pain in 546 school-adolescents, 51.3% of those aged from 14 to 17 years had low back pain complaints, three months previous to the survey date^19^. A Lebanese study reported that almost 87% of the adolescents were diagnosed with musculoskeletal neck pain ^20^. A recent Iranian survey reported that neck, shoulders and low back pain accounted for 35.3%, 26.1% and 33% of all school-adolescents aged from 12 to 14 years ^15^. A previous survey conducted in Central East of Tunisia in 2002 showed that the prevalence of low back pain was 28.4% among school adolescents ^21^, which was much lower than our rate. The discrepancy between our and previous studies might be related to the difference in definition of symptomatic cases, the social and cultural differences between populations, time of exposure and psychological factors. The mean rating of the pain severity ranged from 4.3 to 4.8, which was higher than the Iranian rate (from 2 to 3) ^22^. Indeed, other factors may interfere in the self-reported severity pain judgment, including academic pressure, environmental factors and psychological distress among adolescents.

One of the main findings of this study was that the MSP occurred frequently at multiple sites in the study population, which was in line with the findings of previous studies conducted in this regard ^13,23^. In fact, it has been demonstrated that the likelihood of experiencing synchronized neck and low back pain was relatively high among adolescents for both genders and yielded 17.7% ^13^.

The study highlighted the multi-factorial nature of MSP in school-adolescents. Our results supported findings in other studies that have found that girls were more likely to complaint from symptoms in neck and shoulders than boys ^15^. Previous Brazilian studies reported that the risk of developing a MSP was 10 to 50% higher among girls as compared to boys ^24,25^. Possible explanations for these results may be the earlier female puberty and its accompanying hormonal changes, as well as their anatomical and functional characteristics compared to males. Moreover, it has been reported that boys always have a higher pain threshold than girls ^26^; thus, it is more socially acceptable for women to show their symptoms and feelings because of both societal and educational factors. In our study, older age was significantly associated with neck and low back pain. Previous researches reported that this association remain debatable; a previous European review demonstrated that the low back pain odds ratio increased substantially from 2.79 in the 10-12-year age group to 16.5 in the 16-20-year age group ^27^. On the other hand, a systematic review reported that older age does not increase the likelihood of developing MSP ^28^. Our findings might be linked to higher school grade with advanced age, which has been also proved as a risk factor of neck and low back pain. This result might be closely related to the psychological pressure placed on terminal-years secondary school-adolescents due to firstly, an increase in academic overload and secondly, to sedentary states and the reduction of physical activity caused by the studies burden for this age-group. It was interesting to note that BMI>25Kg/m^2^ was significantly associated with higher risk of neck shoulders and low back complaints and was an independent risk factor of shoulders pain. Similarly, Dianat et al. reported that a BMI<17.22 was an independent protective factor of neck pain occurrence among children and adolescents, while no significant association with shoulders pain was reported ^22^. Therefore, it was not surprising to find that a weekly regular physical activity decreased significantly the likelihood of MSP in school-adolescents. In this same point of view, previous studies emphasized on the role of increasing weekly frequency of exercise in decreasing MSP ^29,30^.

Another relevant finding was the association between time spent watching TV, playing videogames and using computer with the MSP. A previous study showing that watching TV for more than eight hours per day was a risk factor for back pain ^30^. Similarly, it has been reported that watching TV more than two hours per day multiplied by two the risk of low back pain occurrence in adolescents ^31^. Improperly sitting while using electronic devices in inappropriate posture for a long period throughout the day predisposed to higher levels of general discomfort, pain and fatigue. Act of sitting on awkward posture, results in increased intradiscal pressure, disc malnutrition and can endanger the integrity of the musculoskeletal system ^32^. High-quality evidence reported in a previous metanalysis suggested that low socioeconomic status was a risk factor for onset of MSP ^28^. These findings were consistent with our results suggesting the statistical association of living within low-or middle-income families and the protective role of parents high educational level with MSP occurrence.

It was also of particular interest to note the effect of classroom furniture and lay out design on several body regions. This finding highlighted the need for specifically designed interventional programs and effective speciﬁc ergonomic interventions aiming at improving physical factors in the school environment. The final statistical model confirmed that the seat backrest height and the seat-to-(black) board distance were independently associated with low-back pain and that a too low desk was an independent risk factor of neck pain. Moreover, several features of classroom furniture including seat and desk dimensions had significant associations with the occurrence of musculoskeletal complaints in univariate analyses. Using an inappropriate desk may force school-adolescents to lift their arms, which may cause more muscular load pain and discomfort. Similarly, previous studies showed that difficulty in viewing the (black)board and too much homework were independent risk factors of low back pain ^6^. The same findings were consistent with previous researches reporting a positive association between MSP and the chair height being too low ^33^, the backrest of the chair being too curved and the desk height being too low ^34^. Furthermore, schoolbag to body weight>10% was independently associated with shoulders and low back pain. Schoolbag weight has received much attention in recent years in the development of musculoskeletal pain in school-adolescents. This result was in agreement with previous studies ^15^, suggesting that there is an increased risk of back pain occurrence when schoolbag to body weight ratio grew by 1%^10^. Most international standards accept a satchel weighing 10% to 15% of the body weight of the child ^35^. However, schoolbag weight may not alone sufﬁciently describe the needs of the musculoskeletal systems of school-adolescents, other factors might be taken into account: carrying school supplies over a long period of time each day on an asymmetric way might cumulatively enhance the risk of discomfort and musculoskeletal fatigue occurrence, as already described in the literature ^15,19,22,30^. This result can be engendered by the torque-side slope generated on the spine when heavy weight is carried above only one shoulder, and is significantly reduced when it is carried above both shoulders ^36^. Therefore, there is an utmost need for educational program policies to adhere to the reference values of schoolbags according to the anthropometric needs of school-adolescents.

It was worthy to note that neck, shoulders and low-back pain had serious adverse outcomes on medical care requirements, daily life performance and social life in adolescents. The relationship between negative emotional symptoms operationalized as emotional problems, anxiety symptoms, or depressive symptoms and MSP has been previously illustrated ^28^. A previous study showed that parents of school-adolescents with neck pain announced that their children’s complaints had negative effects on their school grades as well as on their psychological behaviors: they preferred to isolate themselves from the social life and become easily irritable ^20^. Previous reports showed that 15.5% of school-adolescents reported that back pain disallowed them to perform daily activities ^30^ and that 24.2% reported that the pain resulted in sleep disorders and required specialized medical care ^19^.

Our original study enlightened the extent of MSP among adolescents and highlighted their major risk factors. However, these results remain hypothetic: Firstly, the cross-sectional study design is useful for characterizing the prevalence of a condition or a risk factor in a study population, unless we can assure temporality. Secondly, another limitation of the cross-sectional studies is the periodic variation of exposure, such as a particularly stressful period, which may affect psychological and somatic health complaints among adolescents. Moreover, data analysis in our study was based on subjective scales, relying on the accuracy and reliability of self-reporting, which can over or underestimate the real burden of MSP among adolescents.

## Conclusion

Our study provided original results illustrating the magnitude of neck, shoulders and low-back pain issues among adolescents in secondary school. The prevalence of these complaints was substantially high among adolescents and their associated risk factors were numerous, including sociodemographic factors, leisure activities and classroom furniture. MSP may lead to serious outcomes threatening the physical performance as well as the social life of adolescents. Once the several surrounding risk factors are well understood, an ergonomic specific and behavior-based school program is urgently needed, which aims at revising the school environment and addressing effective preventive strategies to where they are actually needed. Furthermore, multi-disciplinary approach involving teachers, policy makers and health care professionals is mandatory in order to raise awareness on MSP and its serious consequences in adolescents, by running regular preventive and educational campaigns.

## Acknowledgements

We would like to thank the Regional Directory of Education, the Tunisian Ministry of Education and the school authorities for their active collaboration.

## Conflict of interest statement

All authors declare no conflict of interest.

## Funding

None.

## Highlights

- Neck, shoulders and low back complaints were simultaneously evaluated in this study
- High prevalence of neck, shoulders and low back pain in Tunisian adolescents
- School-furniture and lay out design were associated with musculoskeletal pain
- Exposure to electronic devices was predisposing to high risk of musculoskeletal pain
